# Effect of General Anesthetic Agents on Microglia

**DOI:** 10.14336/AD.2023.1108

**Published:** 2024-05-07

**Authors:** Yanchang Yang, Wenxin Hang, Jun Li, Tiantian Liu, Yuhan Hu, Fuquan Fang, Dandan Yan, Patrick M. McQuillan, Mi Wang, Zhiyong Hu

**Affiliations:** ^1^Department of Anesthesiology, The First Affiliated Hospital, Zhejiang University School of Medicine, Hangzhou, China.; ^2^Department of Anesthesiology, Shulan (Hangzhou) Hospital Affiliated to Zhejiang Shuren University Shulan International Medical College, Hangzhou, China.; ^3^Department of Anesthesiology, Ningbo Women and Children's Hospital, Ningbo, China.; ^4^Cell Biology Department, Yale University, New Haven, CT, USA.; ^5^Department of Anesthesiology, Penn State Hershey Medical Center, Penn State College of Medicine, Hershey, PA, USA.

**Keywords:** microglia, general anesthetic agent, neuroinflammation, central nervous system, postoperative cognitive dysfunction

## Abstract

The effects of general anesthetic agents (GAAs) on microglia and their potential neurotoxicity have attracted the attention of neuroscientists. Microglia play important roles in the inflammatory process and in neuromodulation of the central nervous system. Microglia-mediated neuroinflammation is a key mechanism of neurocognitive dysfunction during the perioperative period. Microglial activation by GAAs induces anti-inflammatory and pro-inflammatory effects in microglia, suggesting that GAAs play a dual role in the mechanism of postoperative cognitive dysfunction. Understanding of the mechanisms by which GAAs regulate microglia may help to reduce the incidence of postoperative adverse effects. Here, we review the actions of GAAs on microglia and the consequent changes in microglial function. We summarize clinical and animal studies associating microglia with general anesthesia and describe how GAAs interact with neurons via microglia to further explore the mechanisms of action of GAAs in the nervous system.

## 1. Introduction

Since William T. Morton's successful demonstration in 1846 that ether inhalational anesthesia could eliminate pain, the development of anesthetics and the use of general anesthesia in the intervening decades have greatly contributed to the advancement of surgery and other invasive procedures. General anesthetic agents (GAAs) provide ideal desired clinical effects, such as unconsciousness, amnesia, analgesia, and immobility [1]. GAAs exert pharmacological effects by selectively acting on different parts of the brain and/or neural pathways (e.g., thalamo-cortical pathway, cortico-cortical pathway), and their main targets include various receptors, ion channels, enzymes, and other proteins. Of these targets, ion channels are the predominant class, including ligand-gated ion receptors such as gamma-aminobutyric acid (GABA), glutamate, and acetylcholine receptors; voltage-gated ion channels including sodium, calcium, and potassium channels; and numerous second messenger-coupled channels [2, 3]. Because the importance of glial cells in the central nervous system (CNS) is gradually being recognized, GAAs have been determined to also act on glial cells rich in various receptors and ion channels [4]. This interaction affects the proliferation, apoptosis, secretion, and regulatory functions of microglia, thereby affecting neurons [5].

Microglia account for 10%-15% of all glial cells [6]. Microglia are the innate immune cells of CNS, involved in the growth and development of CNS, homeostasis formation, and CNS disorders. They can also respond to specific factors such as injury or infection by transforming the phenotype of microglia to perform inflammatory functions [7]. Microglia express multiple receptors for neurotransmitters and neuromodulators including ionotropic and metabotropic glutamate, GABA, adenosine triphosphate (ATP), adenosine, acetylcholine, histamine, and other receptors [8]. Some of these receptors are targets of GAAs. Their intracellular signaling cascade reaction is very important for microglial activation, affecting the detection, elimination, and repair of microglia. In addition to receptors that sense physiological occurrences, microglia express a variety of pattern recognition receptors (PRRs) including toll-like receptors (TLRs), NOD-like receptors, and C-type lectin receptors, which can transmit inflammatory information by recognizing pathogen-associated molecular patterns (PAMPs) or tissue damage-associated molecular patterns (DAMPs) [9]. By recognizing anesthetic drug-sensitive PRRs, GAAs also promote changes in microglial cell type and function and increase or mitigate the release of inflammatory factors [10].

Microglia have recently gained attention as an important target for GAAs. Recent studies have shown that microglial cell ablation can significantly reduce the sensitivity of mice to anesthetics, which is closely related to P2Y12 receptor-mediated signal transduction and expands the non-immune effects of microglia [11]. GAAs have a dual effect on microglia: (a) they inhibit microglial activation and promote polarization toward anti-inflammatory phenotypes to improve cognitive function by exerting anti-neuroinflammatory effects and (b) long-term, high-dose drug exposure or exposure to GAAs during the critical developmental windows (neonatal and older individuals) promotes microglial activation, leading to brain degeneration, neuronal damage, cognitive dysfunction, and neurodegenerative disease. Therefore, it is important to explore the effects of GAAs on the phenotype and function of microglia and to determine how they are involved in microglia-induced neuroinflammation. These findings will not only help to improve the understanding of the mechanism of the effects of GAAs on the brain, but also help to prevent microglia-associated cognitive deficits and neurodegenerative disease and at the same time provide useful suggestions for the selection and use of GAAs in clinical practice.

In this review, we discuss how GAAs act on microglia and the consequent changes in microglial cell function, summarize clinical and animal studies associating microglia with general anesthesia, and explore the potential mechanisms of action of GAAs on the nervous system via microglia.

## 2. Evidence from Preclinical Studies

### 2.1 Clinical Studies

Microglia are regulated by clinical doses of GAAs. In human fetal prefrontal cortex mixed cell cultures, clinically relevant doses/concentrations of both propofol (20 μΜ, 6 h) and sevoflurane (3.3%, 6 h) promoted "microgliosis", and sevoflurane also reduced microglial transcription [12]. The use of GAAs is an important risk factor for perioperative neurocognitive dysfunction (PND) because the microglial activation and neuroinflammation caused by GAAs plays an important role in the pathogenesis of PND [13]. Expression of inflammatory factors including interleukin (IL)-5, IL-6, IL-8, monocyte chemoattractant protein-1 (MCP-1), macrophage inflammatory protein (MIP)-1α, and the receptor for advanced glycation end products (RAGE) were significantly increased in plasma of patients with PND after anesthesia and surgery. Furthermore, cerebrospinal fluid cytokines, including interferon-γ, IL-6, IL-8, IL-10, MCP-1, MIP-1α, MIP-1β, IL-8/IL-10, and tumor necrosis factor-alpha (TNF-α) also changed significantly from baseline levels, suggesting inflammatory activity in the CNS [14]. Persistent increases in plasma pro-inflammatory factors such as IL-6, IL-8, and MCP-1 in patients with delirium demonstrate that anti-inflammatory and pro-inflammatory cytokines are closely associated with cognitive impairment and decreased brain function after general anesthesia [14]. Although the pathogenesis of PND is still unclear, changes in cytokines secreted by microglia and cognitive dysfunction of patients in their later years confirm the key role of microglia in the pathogenesis of PND. In addition, nerve injury can induce significant microglial proliferation and microglial activation to release bioactive mediators, thus promoting pain signaling in the spinal cord, with supraspinal levels leading to neuropathic pain [15]. Therefore, the dual regulation of microglia by GAAs may provide a basis for GAAs to enhance/relieve pain.

Inflammatory injury in the early stages of brain development induces microglial activation, which leads to synaptic refinement and synaptic construction; in later stages, defects in plasticity and neuron connectivity are observed [16]. The subsequent promotion or inhibition of stress or other interference conditions can lead to schizophrenia, depression, and anxiety. Patients with severe depression showed synaptic defects and activation of microglia related to brain inflammation, particularly in the anterior cingulate cortex, which is closely related to the regulation of stress and emotion [17, 18]. In patients with severe depression, a single intravenous dose of ketamine could significantly alleviate the symptoms of depression for 24 h, , and the treatment gains associated with ketamine exhibit short-term effect (2 weeks), which is closely related to transformation of the microglial phenotype [19]. Because GAAs can induce activation of microglia and their transformation to different phenotypes, this may provide evidence for the occurrence or relief of depression or other emotional changes in children after repeated exposure to GAAs during brain development (Table 1).

**Table 1:** Summary of the changes in microglia induced by general anesthetic agents in human studies.

GAAs	Experimental subjects	Concentration or dose	Exposure time	Results
**Propofol**	Human fetal prefrontal cortex	20 μΜ	6 h	The proportion of microglia increased by nearly 4 times, without further changes in number after 2 hours of recovery [12].
	Human aged ≥ 55 years; plasma	/	/	Expression of inflammatory factors including IL-5, IL-6, IL-8, MCP-1, MIP-1α, and RAGE were significantly increased in the plasma of PND patients [14].
	Human aged ≥ 55 years; cerebrospinal fluid	/	/	Expression of cerebrospinal fluid cytokines including IFN-γ, IL-6, IL-8, IL-10, MCP-1, MIP-1α, MIP-1β, IL-8/IL-10, and TNF-α changed significantly from baseline levels [14].
**Ketamine**	Human with major depressive disorder and post-traumatic stress disorder	0.5 mg / kg	40 mins	A post-hoc, exploratory analysis suggests that patients with lower SV2A density at baseline may exhibit increased SV2A density 24 hours after ketamine [17].
**Sevoflurane**	Human fetal prefrontal cortex	3.3%	6 h	After 2 hours of recovery, microglia population increased by 4 times from the baseline and sevoflurane decreased microglia transcriptional similarity [12].

Abbreviations: GAAs, general anesthetic agents; MCP-1, monocyte chemoattractant protein-1; MIP, macrophage inflammatory protein; RAGE, receptor for advanced glycation end products; PND, perioperative neurocognitive dysfunction; SV2A, synaptic vesicle protein 2A; IL, interleukin; TNF, tumor necrosis factor.

### 2.2 Animal Studies

Many in vivo and in vitro experiments have shown that commonly used GAAs, including propofol, ketamine, sevoflurane, isoflurane, and desflurane, can inhibit activation of microglia and related inflammatory pathways and promote M2 polarization to improve cognitive function. In contrast, long-term or high-dose exposure to GAAs during critical stages of brain development and functional decline can promote the occurrence of inflammatory reactions and increase the risk of GAAs inducing PND (Table 2). Microglial activation is closely related to changes in cognitive function. In animal experiments related to PND, the degree of microglial activation is often used as an important index to judge neuroinflammation and neuronal damage [20], while depletion of microglia or interruption of microglial activation can alleviate neuroinflammation and cognitive function decline [21].

After nerve injury, upregulation of microglial cell surface receptors (e.g., purinergic receptors, TLRs, chemokine receptors) mediates p38 mitogen-activated protein kinases (MAPKs) and phosphorylation of extracellular signal-regulated kinases (ERK) 1/2 and 5, thereby increasing the synthesis and release of microglial factors that mediate pain (e.g., TNF-α, IL-1β, IL-6, BDNF, prostaglandin E2) [22]. The modulatory effect of GAAs on the above pathways seems to influence the neuropathic pain process (Table 2).

Lipopolysaccharide (LPS) is a common laboratory microglia activator. After exposure to LPS in the early developmental stages of mice, they exhibit neurochemical changes similar to autism spectrum disorders, with neuronal atrophy in the prefrontal cortex and hippocampus [23]. The initial inflammation induced by LPS as well as mood disorders can be prevented by ketamine or antidepressant administration. In addition, ketamine attenuated LPS-induced depression-like behavior by increasing alpha-amino-3-hydroxy-5-methyl-4-isoxazolepropionic acid (AMPA) signaling [24]. Thus, GAAs can participate in mood disorder regulation via microglia.

## 3. Impact of GAAs on Microglial Activation and Phenotypic Changes

### 3.1 The function of microglia

As resident macrophages in the CNS, microglia perform disease modification and regulation of other glial cell groups (including astrocytes and oligodendrocytes) [25]. Microglia are highly dynamic and reactive under normal conditions, participating in development, balance maintenance, and disorders of the CNS. Microglia release trophic factors required for the formation of neuronal circuits, such as insulin-like growth factor-1, to promote their survival. Early postnatal accumulation of microglia in subcerebral and callosal axon fibers may be involved in the action and support of these fibers [7]. In addition, neurotrophic factors such as brain-derived neurotrophic factor (BDNF), nerve growth factor, epidermal growth factor, platelet-derived growth factor, and other neurotrophic factors released by microglia during the early developmental stages contribute to neuronal growth and development [6]. When an organism is injured, microglia are activated before monocyte-derived macrophages reach the site of inflammation, promoting the synthesis and secretion of cytokines, chemokines, prostaglandins, and other immunomodulatory molecules, and are involved in monocyte recruitment to the CNS [26]. Microglia not only maintain development of the CNS, but also contribute to the maintenance of synaptic and brain neural network homeostasis by participating in the induction of programmed cell death and phagocytic cell fragmentation [7]. Microglia selectively prune redundant and excessive neurons that are not involved in neuronal circuit formation and regulate synaptic activity and plasticity [27]. Abnormal or prolonged activation of microglia can lead to brain degeneration, neuronal damage, and neurodegenerative disease [28]. Indeed, many risk genes for CNS diseases revealed by genome-wide association studies (including Alzheimer's disease and Parkinson's disease) are expressed by microglia [6].

**Table 2:** Summary of the changes in microglia induced by general anesthetic agents in various studies.

GAAs	Experimental subjects	Exposure period	Concentration or dose	Exposure time	Mechanisms
**Propofol**	BV2 cells	/	50 μmol/ L	2 h	Inhibited the NMDA receptor and LPS-mediated Ca2+ accumulation, and attenuated LPS-induced phosphorylation of CaMK II, ERK1/2, and NF-κB [55].
	BV2 cells	/	25 μmol/L	2 h	Maintained the intracellular Ca2+ hemostasis and activated JAK1/STAT-3 pathway [58].
	BV2 cells; primary microglia	/	10-100 μM	/	Inhibited microglia inflammation by targeting TGM2/NF-κB signaling [60].
	Mice; primary microglia; BV2 cells	P1	50 μM	48 h	Inhibited microglial activation via miR-106b/PI3K/Akt axis. And high dose of propofol (100 μM) exhibited strong cytotoxicity [41].
	BV2 cells	/	25-100 μM	3 h	Attenuated hypoxia-induced neuroinflammation by inhibiting oxidative stress and NF-κB/Hif-1ɑ signaling [62].
	Mice; BV2 cells	4 w	5 mg/kg; 25-100 μM	/	Attenuated neuroinflammation (TNF-ɑ, IL-6, and IL-1β expression) through inhibition of the ERK1/2/NF-κB pathway [67].
	Rats	P7	50-150 mg/kg	/	Edaravone alleviated propofol-induced neural injury in developing rats by BDNF/TrkB pathway [93].
	HMC3 cells	/	1000 nM	30 mins	Suppressed IL-6 and IL-8 production as wells as the phosphorylation of p38 MAPK and IκB and the translocation of NF-κB [145].
	BV2 cells	/	30 μM	4 h	Inhibited augmentation of TLR4 expression and further upregulated LPS-induced inactivation of GSK-3β [65].
	Rats	P7	20 mg/ kg	2-6 h	Activated Fas/FasL-mediated extrinsic and Bcl-2-dependent intrinsic apoptotic pathways, resulting in caspase-8 and caspase-9 activations [140]
	Rats	18-20 months	200 mg/kg	For 6 days	Induced neuronal damage and cognitive impairment by activation of NF-κB pathway and NLRP3 inflammasome [88].
	BV2 cells	/	30 μM	10 h	Reduced microglia activation and neurotoxicity through inhibition of EVs release [79].
	Rats; primary microglia	/	40 μM	24 h	Decreased Cx43, Cx43 phosphorylation, and CaV3.2 levels in microglial cells [82]
	BV2 cells	/	25-100 μM	24 h	Suppressed the neuroinflammatory response of microglia to LPS through the regulation of the miR-155/SOCS1 pathway [71].
	BV2 cells; primary microglia	/	10-100 μM	/	Targeted the miR-221/-222-IRF2 axis to prevent microglial activation in two independent cell models [75].
	Rats	20 months	15 mg/kg; 1 mg/kg/min	45 mins	Reduced inflammation by up-regulating miR-223-3p, thereby reducing POCD.
**Ketamine**	BV2 cells	/	10 μg/ mL	2 h	Inhibited NMDA receptors, attenuated Ca2+ levels, and inhibited phosphorylation of CaMK II phosphorylation, NF-κB and nuclear translocation [56].
	Primary cultured microglia	P7	100-500 μM	30 mins	Inhibited ERK1/2 phosphorylation [97].
	HMC3 cells	/	400 nM	24 h	Regulated the type I interferon pathway by nuclear translocation of STAT-3 in human microglia [108].
	Mice	/	20 mg/kg	1 h	Inhibited elevated levels of pro-inflammatory factors in the brains of stressed mice via the TLR4/p38 MAPK pathway and alleviates depression-like behavior [103].
	Human fetal microglial cells	/	25-150 μM	6 h	Induced neuronal apoptosis and promoted pro-inflammatory M1 phenotypes [36].
	Mice; BV2 cells	8-10 w	10 mg / kg	/	Improved depression-like behavior, inhibited the upregulation of HMGB1 and RAGE and the nuclear translocation of HMGB1 [113].
	Mice	2 months; 16 months	7 mg / kg	/	Alleviated POD-Like symptoms and improved abnormal levels of BDNF-TrkB Signaling [105].
	Rats	P7	20 mg/kg	five doses; every 90 mins	Blockade of the NLRP3/caspase-1 axis attenuated ketamine-induced hippocampus pyroptosis and cognitive impairment in neonatal rats [101]
**Esketamine**	Rats	Adult	5 mg/kg	/	Alleviated inflammatory response, phosphorylation of NF-κB and microglia activation in striatum and PAG [98].
	Mice	7 w	5 mg/kg	/	Alleviated inflammatory actions through BDNF-TrkB signaling pathway and inhibited POD-induced activating of NF-κB pathway [106].
**Sevoflurane**	H4 cells	/	4.1%	6 h	Increased the levels of IL-6, the nuclear levels of NF-κB and the transcription activity of NF-κB [37].
	Mice; primary microglial cells	P1-2	2%/4%	12 h	Abolished anti-inflammatory M2 microglia marker genes and proteins expression Arg1, Ym1, and IL-10 [38].
	Mice; primary cortical microglia	8-10 w; P1	2.5%	1 h	Protected experimental ischemic stroke by enhancing anti-inflammatory M2 microglia polarization through GSK-3β/Nrf2 pathway [40].
	Rats	P85-99	3%	2 h	Enhanced the release of cytokines and the activation of intracellular NF-κB signaling [119].
	Rats	P7	2%/3%	4 h/day; 3 days	Induced inflammation of microglia in hippocampus by inhibiting Wnt/β-Catenin/CaMKIV pathway [153].
	Mice	4 months	5%	50 mins	Alleviated the activation of NLRP3 inflammasome and microglia in the hippocampus [154].
	Mice; primary cortical microglia	P6; P2	3%	2 h; 3 days	Induced tau exit from neurons upon phosphorylation, travel through both EVs and non-EVs routes, and then enter microglia, leading to generation of IL-6 [129].
	Mice	P6	3 h	2 h; 3days	Induced Tau phosphorylation and cognitive impairment in neonatal mice [133].
**Isoflurane**	H4 cells	/	2%	6 h	Increased the levels of IL-6, the nuclear levels of NF-κB and enhanced the transcription activity of NF-κB [37].
	Rats	20 months	1.5%	4 h	Activated the canonical NF-κB pathway and induced hippocampal interleukin-1β elevation and the resultant cognitive deficits [120].
	Rats	P1; 8w	0.7%	1 h	Abated ROS-activated MAPK/NF-κB signaling to repress ischemia-induced microglia inflammation and brain injury [155].
	BV2 cells	/	3%	24 h	Reduced TREM2 expression and caused microglia activation, neuroinflammation, and oxidative stress [140]
	Mice	6-8 months; 14 months	1.5%	2 h	activated NLRP3-caspase-1 pathway and increased the secretion of IL-18 and IL-1β [102].
**Dexmedetomidine**	Mice	16 months	20 μg/kg	/	Alleviated sevoflurane-induced neuroinflammation and neurocognitive disorders by suppressing the P2X4R/NLRP3 pathway [156].
**Etomidate**	Mice	18 months	20 mg/kg; 8 mg/kg	/	Acticated microglia during the early pathological stage of PND creates an inflammatory environment and stimulates A1-specific astrocyte responses during the late pathological stage [152].

Abbreviations: GAAs, general anesthetic agents; NMDA, N-methyl-D-aspartic acid; LPS, lipopolysaccharide; CaMKII, calmodulin-dependent protein kinase II; ERK, extracellular signal-regulated kinase; JAK1, Janus Kinase 1; STAT-3, signal transducer and activator of transcription 3; TGM2, transglutaminase 2; P1, postnatal day 1; PI3K, phosphoinositide 3-kinase; Hif-1α, hypoxia inducible factor-1α; BDNF, brain-derived neurotrophic factor; TrkB, tyrosine kinase receptor B; MAPK, mitogen-activated protein kinase; TLR, toll-like receptor; GSK-3β, glycogen synthase kinase-3β; NLRP3, NOD-like receptor protein 3 inflammasome; EV, extracellular vesicle; Cx43, connexin 43; SOCS1, suppressor of cytokine signaling 1; IRF2, interferon regulatory factor 2; POCD, postoperative cognitive dysfunction; HMGB1, high-mobility group box-1 protein; RAGE, receptor for advanced glycation end products; POD, postoperative depression; PAG, periaqueductal gray; TREM2, triggering receptor expressed on myeloid cells-2; Nrf2, nuclear factor E2-related factor 2; CaMKIV, calmodulin-dependent protein kinase IV; P2X4R, purinergic ionotropic 4 receptor; PND, perioperative neurocognitive disorders; IL, interleukin; TNF, tumor necrosis factor; ROS, reactive oxygen species.

### 3.2 Changes in the Phenotype of Microglia

**Figure 1.**
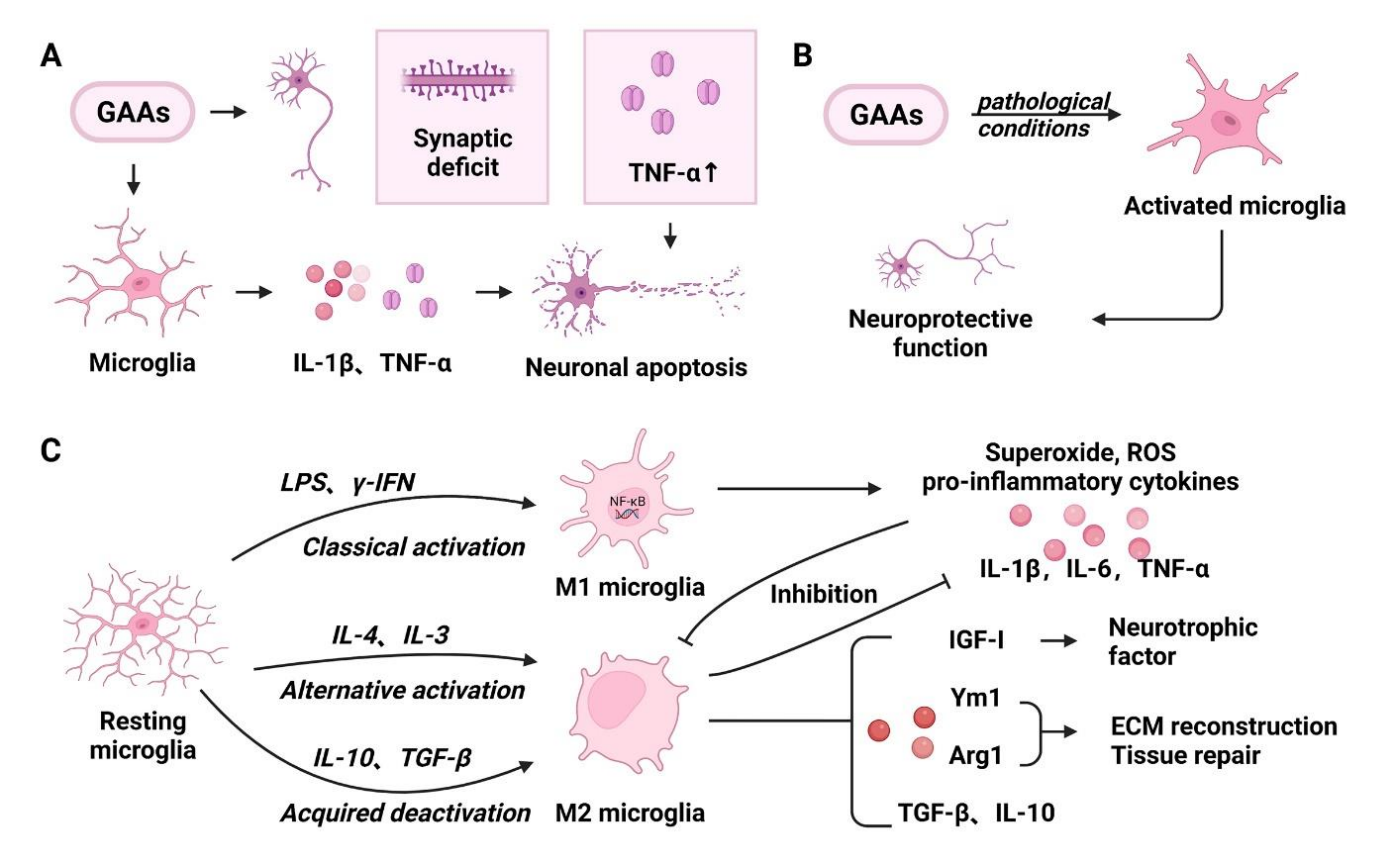
**Functional alterations of microglia after exposure to general anesthetic agents (GAAs)**. (**A**) GAAs activate microglia to release interleukin 1β (IL-1β) and tumor necrosis factor-ɑ (TNF-ɑ) and act on neurons, increasing their TNF-ɑ content and collectively promoting neuronal apoptosis. (**B**) Activation of microglia by GAAs under pathological conditions is neuroprotective. (**C**) Microglia have three states: classical activation, alternative activation, and acquired inactivation. Lipopolysaccharide (LPS) and interferon-gamma (IFN-γ) can activate resting microglia to the M1 type, i.e., the "classical activation" state. The M1 microglia produce reactive oxygen species (ROS) and superoxide and induce the NF-κB pathway to produce pro-inflammatory cytokines such as IL-1β, IL-6, and TNF-ɑ. M2 microglia are alternatively activated and inactivated by IL-4, IL-13, and IL-10 and transforming growth factor-β (TGF-β). M2 microglia induce an anti-inflammatory response by producing TGF-β and IL-10 and enhance the release of neurotrophic factors such as insulin-like growth factor 1 (IGF-1), which promotes neuronal survival. In contrast, production of substances such as arginase 1 (Arg1) and Ym1 aids in extracellular matrix (ECM) reconstruction and tissue repair.

Microglia are both neuroprotective and neurotoxic and are traditionally classified into M1 (classical pro-inflammatory) or M2 (anti-inflammatory) phenotypes (Fig. 1). Cytokines produced in response to LPS, γ-interferon, and T helper 1 cells can cause dormant microglia to be activated to the M1 phenotype [29], releasing pro-inflammatory mediators such as IL-1β, IL-6, TNF-α, C-C motif ligand 2, and reactive oxygen species [30, 31]. This immune response is triggered by PAMPs or proteins released from damaged neurons to induce TLR activation, which damages the corresponding neurons [32]. In contrast, the anti-inflammatory cytokines IL-4 and IL-13 induce polarization of microglia toward the M2 phenotype [33]. By producing transforming growth factor-β and IL-10 to induce anti-inflammatory responses, microglia inhibit M1 macrophage-mediated inflammation and exert neuroprotective functions to promote neural remodeling and repair [34]. The M1/M2 transition may play an important role in maintaining the balance between promotion and regression of neuroinflammation in the CNS. Furthermore, it is worth mentioning that the simple M1/M2 classification is challenged by the updated kaleidoscope view that has been proposed for the microglial phenotype [35].

GAAs induce the conversion of microglia to pro-inflammatory (M1) and anti-inflammatory (M2) phenotypes (Table 2). Ketamine promotes the conversion of microglia to the M1 phenotype [36]. Exposure to sevoflurane (4.1%, 6 h) resulted in an increase in IL-6 and NF-κB activities in glioma cells [37]. These studies suggest that GAAs can induce neuroinflammation. Similarly, in primary mouse microglia, sevoflurane (2%/4%, 12 h) pretreatment significantly attenuated the induction of characteristic M2 marker genes as well as Arg1, Ym1, and IL-10, which are mediated by IL-4, inhibited expression of suppressor of cytokine signaling (SOCS) 1, and resumed SOCS3 expression, thus inhibiting the IL-4-induced activation of M2 phenotypic microglia [38]. In addition to the important protective role of M2 microglia against inflammation and injury, they can increase the number of neurons and the overall neuronal length [39]. Therefore, the sevoflurane-induced inhibition of M2 microglial polarization also indicates that sevoflurane can be harmful to the integrity of neurons and cause neurotoxicity. In addition, GAAs induce activation of the anti-inflammatory M2 microglial phenotype under the same conditions. A subanesthetic dose of sevoflurane (2.5%, 1 h for 5 days) mediated M2-type microglial polarization via glycogen synthase kinase (GSK)-2β phosphorylation and Nrf3 activation, promoting neuroprotection by inducing polarization of microglia toward an anti-inflammatory phenotype in ischemic disease [40]. Propofol also has a significant neuroprotective effect. Exposure to high concentrations of propofol not only attenuated microglial activity but also increased microglia apoptosis [41]. Thus, phenotypic transformation of microglia and the neurotoxic or neuroprotective effects induced by different anesthetic drugs may depend on the disease model, concentration, and/or exposure time.

### 3.3 TLR Signaling in Microglial Activation

TLRs play an important role in establishing innate immunity. Their role as PRRs can be triggered by structural motifs of PAMPs, which are expressed by various pathogens such as bacteria and viruses [42]. In addition, TLRs mediate the recognition of DAMPs produced by the host itself. TLRs activate pro-inflammatory immune responses by recognizing various modes/molecules from pathogens and the host as well as activating NF-κB and other inflammatory pathways and transcription factors that lead to the synthesis of pro-inflammatory molecules [43]. Microglial activation is related to upregulation of TLRs, and TLR knockout in mice prevents microglial activation [44]. TLR4 is abundantly expressed in microglia and participates in a series of inflammatory pathways including those related to NF-κB, MAPK, and phosphatidylinositol 3-kinase (PI3K)/Akt [45].

**Figure 2.**
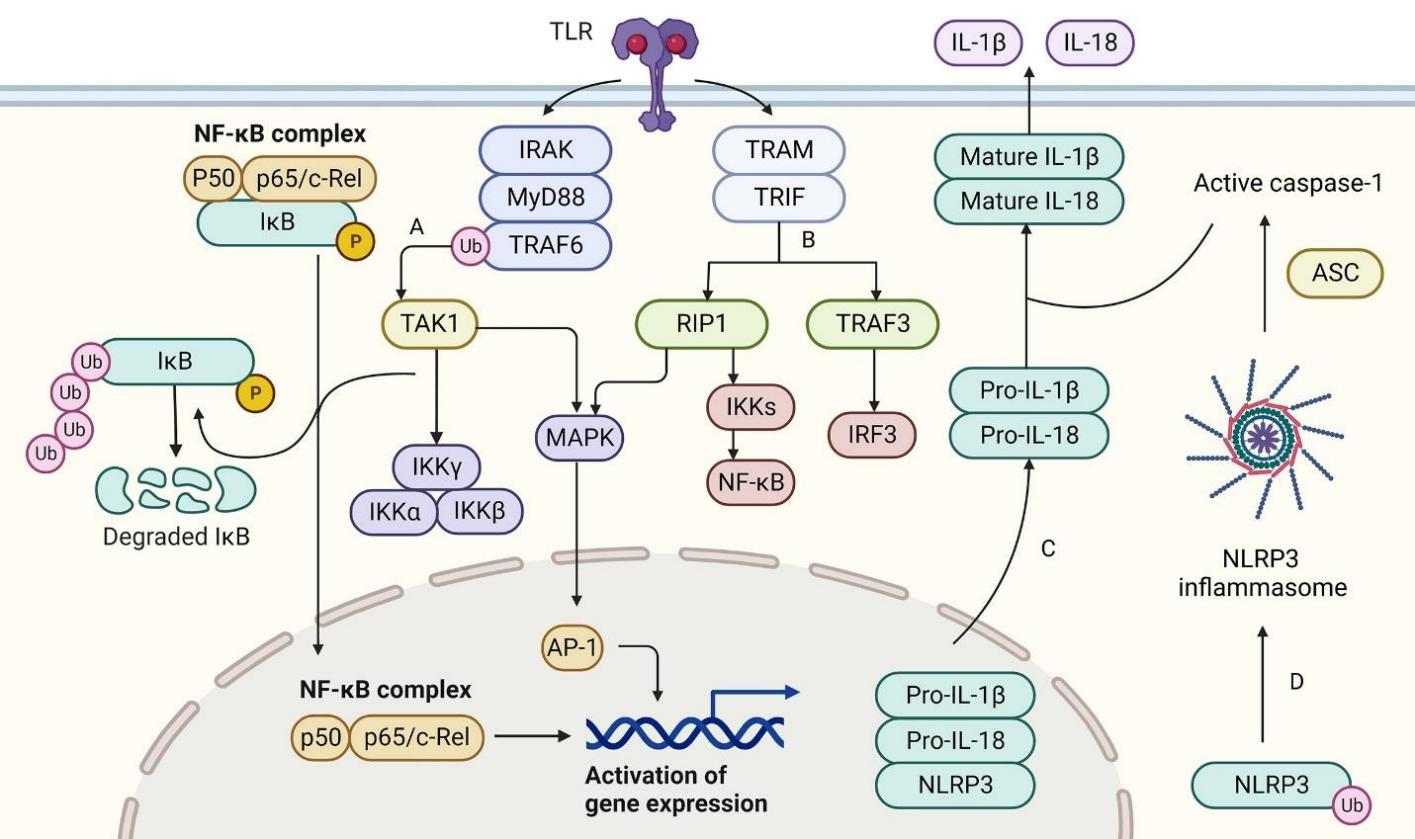
**Toll-like receptors (TLRs) and their associated inflammatory pathways**. Lipopolysaccharide generates a signaling cascade through two TLR adapters, MyD88 and TIR domain-containing adaptor, inducing interferon-β (TRIF). MyD88 interacts with the active TLR homodimer complex through its toll-interleukin receptor (TIR) domain and recruits interleukin-1 receptor-associated kinase (IRAK), really interesting new gene (RING)-domain E3 ubiquitin ligase, and TNF receptor-associated factor (TRAF6). Ubiquitination of TRAF6 itself and the transforming growth factor beta-activated kinase1 (TAK1) protein kinase complex results in TAK1 activation, which leads to further transduction of inflammatory messages via the downstream IkappaB kinase (IKK) and mitogen-activated protein kinase (MAPK) pathways. (**B**) TRIF can interact with TLRs through translocation-associated membrane protein (TRAM) to activate the transcription factors interferon regulatory factor 3 (IRF3), NF-κB, and AP-1 through recruitment of TRAF3 and RIP1, which in turn induces activation of NF-κB and MAPK as well as inflammatory factors. (**C**) The active NF-κB promotes transcription of NF-κB-dependent genes, such as NOD-like receptor protein 3 inflammasome (NLRP3), pro-interleukin (IL)-1β, and pro-IL-18. (**D**) The inflammasome activation signal is provided by NLRP3 agonists that activate NLRP3 to trigger inflammasome assembly and mature IL-1β secretion.

LPS generates a signaling cascade through two TLR adapters, MyD88 and toll-interleukin receptor domain-containing adaptor inducing interferon-β (TRIF) [46]. The MyD88-dependent TLR pathway is closely associated with expression of the proinflammatory response. MyD88 interacts with the active TLR4 homodimer complex through its toll-interleukin receptor domain and recruits IL-1 receptor-associated kinase, really interesting new gene (RING)-domain E3 ubiquitin ligase, and TNF receptor-associated factor (TRAF) 6 [32]. Ubiquitination of TRAF6 itself and the transforming growth factor β-activated kinase1 (TAK1) protein kinase complex results in TAK1 activation, which leads to further transduction of inflammatory messages via the downstream IκB kinase and MAPK pathways [47]. In addition, TRIF can interact with TLR4 through translocation-associated membrane proteins to activate the transcription factors interferon regulatory factor 3 (IRF3), NF-κB, and AP-1 through recruitment of TRAF3 and receptor-interacting protein kinase 1, which in turn induce activation of NF-κB, MAPK, and other inflammatory factors [48] (Fig. 2).

TLRs are important inflammation-associated receptors in microglia that act as upstream signals to mediate activation of a variety of inflammation-associated signaling pathways including the NF-κB, MAPK, and PI3K/AKT pathways. These receptors are GAA targets and provide the basis for the regulatory response of GAAs (Table 2). TLR overexpression is associated with age-related cognitive decline. TLR4 levels were significantly elevated in the hippocampus of aged rats compared with those in young rats. In particular, microglia overexpressing TLR2/TLR4 could increase neuroinflammation, causing hippocampus-dependent memory impairment upon stimulation [49]. The more widely studied NF-κB pathway, the inflammasome pathway, and the signal transducer and activator of transcription 3 (STAT-3) pathway all drive production of pro-inflammatory factors. The activation of TLRs and related inflammatory pathways in microglia by GAAs may be closely linked to cognitive impairment induction by GAAs [42, 50].

## 4. Mechanisms Underlying GAA-Microglia Interactions

### 4.1 Propofol

Propofol is a common intravenous anesthetic used clinically to potentiate GABAA receptors and block N-methyl-D-aspartate (NMDA) receptors. Previous studies confirmed that GABAA (ionotropic) and GABAB (metabotropic) receptors are expressed in mice, primary rat microglia, and primary human microglia [51-53]. GABA receptor agonists inhibit LPS-induced increases in TNF-α and IL-6 levels in human microglia. Propofol-induced unconsciousness and hypnotic effects may be attributed to GABAA receptor activation, and GABAA receptor antagonists may reverse the effects of propofol [54]. NMDA receptors are expressed in BV-2 cells (a microglia cell line). In addition, propofol reduces calcium accumulation and inhibits phosphorylation of calcium/calmodulin-dependent protein kinase II (CaMKII) by inhibiting NMDA receptors [55, 56]. This protective function is related to the inhibitory effect of propofol on phosphorylation of the NR1 subunit of the NMDA receptor, which is mediated by a signal for protein phosphatase 2A activation [57]. All of these receptors constitute upstream signaling for microglial activation by propofol.

The signaling levels of calcium play a key role in determining whether microglia can induce neuroinflammation. CaMKII activates the downstream molecules ERK and NF-κB to mediate inflammation and injury. Propofol regulated CAMKIIα in a hypoxia model by inhibiting calcium overload to maintain homeostasis and reduced ERK and NF-κB phosphorylation to prevent microglia-induced inflammation and apoptosis [58]. Transglutaminase 2 (TG2), a calcium-dependent enzyme, is involved in pathophysiological processes such as cell growth, differentiation, and apoptosis by regulating GTPase, cell adhesion, protein disulfide isomerase, kinases, and the cell scaffold [59]. TG2 is closely related to the regulation of glial cell function, and high expression levels promote microglial activation by activating NF-κB signaling. Propofol targets the TG2/NF-κB signaling pathway to inhibit microglial inflammation. These findings demonstrate that TG2 may be a target of propofol in producing anti-inflammatory effects [60].

The transcription factor NF-κB is an important regulator that is involved in the pro-inflammatory response of microglia by promoting the expression of several inflammatory factors. Activation of the IkappaB kinase (IKK) pathway induces the IKK complex to phosphorylate NF-κB repressor proteins, which leads to translocation of the p50/p65 NF-κB heterodimer to form nuclear NF-κB, thus inducing pro-inflammatory gene expression [61]. Propofol exerts neuroprotective effects by inhibiting the NF-κB/hypoxia-inducible factor-1α signaling pathway and suppressing the transcription of inflammation and oxidative stress-related genes to attenuate tissue damage [62]. In addition to targeting the NF-κB pathway, propofol acts on signaling molecules upstream of this pathway, such as TLRs, as well as MAPK signaling to inhibit microglial activation (Table 2).

**Figure 3.**
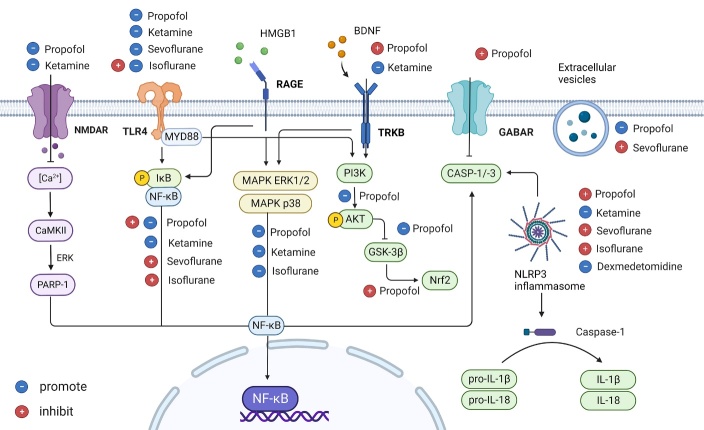
Potential signaling pathways involved in the mechanism of microglial activation by anesthetic agents.

GSK3, a multifunctional protein kinase, is involved in a variety of cellular functions, including cell proliferation, apoptosis, embryonic development, and metabolism. GSK-3β plays a central role in the canonical Wnt pathway by regulating phosphorylation and degradation of β-catenin. GSK-3β is constitutively active as a downstream target of the PI3K pathway, which can be inactivated by phosphorylation of activated Akt, and promotes polarization toward M2-type microglia [63]. Akt, a key mediator of the PI3K pathway, further activates NF-κB to induce inflammation. Inhibition or blockade of the Myd88/PI3K/Akt/NF-κB signaling pathway in microglia attenuates the inflammatory response [64]. Propofol pretreatment downregulated TLR4 expression in BV-2 microglia and induced GSK-3β phosphorylation, which significantly inhibited LPS-induced increases in TNF-α, IL-1β, and IL-10 [65]. Inactivation of GSK-3β inhibits NF-κB p65 transactivation activity, and the MLK3/ Jun amino-terminal kinase (JNK) signaling cascade mediates microglial anti-inflammatory responses. Thus, propofol may exert anti-inflammatory effects through the TLR4/GSK-3β/PI3K/AKT/NF-κB axis (Fig. 3).

TLRs activate the expression of MAPK family members including ERK 1/2, JNK, and p38 proteins. Activation of the MAPK pathway in microglia enhances the release of pro-inflammatory cytokines, which in turn activates the downstream signal NF-κB and promotes the polarization of M1-type microglia to enhance the inflammatory response [66]. Propofol significantly attenuates pain hypersensitivity by interfering with pro-inflammatory microglial activation through inhibition of the MAPK ERK1/2/NF-κB pathway [67]. Despite the weak analgesic effect of propofol, inhibition of this pathway explains the mechanism by which propofol alleviates neuroinflammation and pain to some extent. These studies confirm that the TLR4/MyD88, MAPK, NF-κB, and PI3k/Akt signaling pathway are all involved in the anti-inflammatory mechanisms mediated by propofol via microglia, providing evidence of the benefits of propofol for clinical use.

Microglial activation is related to the over-expression of microRNAs such as miR-22-3p, miR-125b-5p, miR-146b-5p, miR-155-5p, and miR-214-3p [68]. GAAs can significantly affect microRNA gene expression levels in the hippocampus [69]. MiR-155 is an important component of immune regulation and is involved in the inflammatory mediator reaction between macrophages and monocytes. As the target transcript of miR-155, SOCS-1 mainly inhibits the inflammatory process by inhibiting the JAK/STAT pathway [70]. After microglia were exposed to LPS, miR-155 expression was significantly increased and the SOCS-1 level was decreased, which promoted inflammation. Propofol can inhibit the LPS-induced inflammatory response of microglia by regulating the miR-155/SOCS-1 pathway. The anti-inflammatory effect of propofol is weakened after knocking down miR-155 [71]. The C-terminal SOCS box domain interacts with components of the ubiquitin ligase system, which is involved in the degradation of proinflammatory pathway proteasomes including NF-κB and JNK [72]. Regulation of the miR-155/SOCS-1 pathway may be an upstream mechanism by which propofol inhibits NF-κB-related signaling pathways. Furthermore, exposure to propofol suppressed mRNA levels of NF-κB pathway-related genes including Ticam1, Myd88, IRF3, and Nfkb1 in microglia from LPS-treated primary mice culture inhibiting microglia activation via the miR-106b/PI3K/AKT axis [41]. These results demonstrate a novel molecular mechanism of propofol-mediated immunomodulatory effects.

MiR-221 and miR-222 are downstream targets of propofol and are closely associated with microglia activation [73]. Meanwhile, the anti-inflammatory transcription factor Irf2 has been identified as a direct target gene of miR-221/222, binding its 3'-untranslated region to inhibit its translation [74]. Propofol can inhibit microglial activation through the miR-221/222-Irf2 axis [75]. MiR-221 and miR-222 are involved in regulating physiological vascular processes in healthy organisms, yet they also promote a malignant phenotype by mediating the proliferation, differentiation, and invasive capacity of cancer cells [76]. Given the inhibitory effect of propofol on this target, propofol may be beneficial for anesthetic use in cancer patients.

Microglia can secrete large amounts of extracellular vesicles (EVs) that mediate intercellular communication by delivering proteins, nucleic acids, lipids, and cytokines to cells [77]. Increased secretion of EVs during neuroinflammation exacerbates pathogenic processes in the brain [78]. Propofol significantly reduced EV release from immune-activated microglia, and addition of exogenous EVs reversed propofol-mediated anti-inflammatory and neuroprotective effects [79]. This finding suggests that propofol may inhibit microglia-mediated neurotoxic effects by downregulating EV release and decreasing the release of pro-inflammatory factors and activation of pro-inflammatory microglia, which provides a theoretical basis for propofol's anti-inflammatory effects.

In addition to EVs, cell junctions have an important role in signaling. Cell junctions are mainly composed of two protein families, connexins, which form gap junctions (GJs) or hemichannels (HCs), and pannexins, which form pannexons [80]. The former communicates as pore structures by forming channels to connect cells directly to adjacent cells or the environment, while the latter secretes ions or molecules into the extracellular environment to accomplish indirect communication [81]. Connexin 43 (Cx43) can form the GJs and HCs involved in direct intercellular communication. Cx43 is expressed in activated microglia and can exacerbate injury during ischemia by upregulating itself to promote hypoxic injury and propagation of apoptotic signals to adjacent tissues. Hypoxia/reoxygenation injury model studies have shown that propofol protects the brain from ischemia/reperfusion (I/R) injury by downregulating Cx43 expression in microglia and microglial activation [82]. Upregulation of Cx43 after ischemia is considered to promote the transport of cytotoxic molecules (such as TNFα and IL-1β), cell fragments, and dying cells to neighboring cells and tissues and even aggravates cell injury and brain inflammation by transmitting cytotoxic signals through GJs [83, 84]. Regulation of connexin activity in microglia contributes to the reduction of oxidative stress in neurodegenerative diseases and has potential pharmacological promise to delay or halt the progression of these diseases [85]. Therefore, propofol's inhibition of Cx43 may reduce the transmission of inflammatory signals and crosstalk of microglial activation information (such as ATP and TNF-α signaling), thus protecting the brain.

However, propofol not only has a neuroprotective effect by inhibiting inflammation, but also promotes inflammation and activation of microglia in an age- and dose-dependent manner. Although the mechanism is not clear, propofol exhibits pro-inflammatory effects in the developing brain, leading to cognitive impairment in newborn mice [86]. Additionally, repeated exposure to propofol causes upregulation of NF-κB and the NLRP3 inflammasome, resulting in long-term cognitive impairment in the brains of neonatal and aged rats [87, 88]. Studies have shown that targeting the NLRP3 inflammasome signaling pathway in microglia effectively attenuates cognitive deficits in PND and is a potential target for the prevention and treatment of neuroinflammatory diseases [89, 90]. Therefore, intervention in this pathway may be beneficial to prevent propofol-induced inflammatory responses.

BDNF is a key neurotrophic factor involved in neurogenesis and transmission. The mechanism of propofol-induced activation of pro-inflammatory microglia is closely related to downregulation of BDNF. Microglial BDNF binds to neuronal tyrosine kinase receptor B (TrkB), which can activate downstream intracellular cascade responses, including the phospholipase C-gamma, mechanistic target of rapamycin, MAPK, and PI3K pathways [91]. The BDNF/TrkB pathway participates in neuronal growth, survival, and long-term potentiation and enhances BDNF expression. Activation of TrkB signaling plays an important role in hippocampus-dependent learning, memory, and emotional behavior [92]. Activation of the BDNF/TrkB/PI3K/Akt pathway can alleviate nervous system injury caused by propofol [93]. In addition, microglia express TrkB themselves, and BDNF release from microglia promotes their proliferation [94]. Dysregulation of the BDNF/TrkB pathway is commonly observed in CNS diseases such as Alzheimer’s disease, Parkinson's disease, and stroke, and microglial-expressed BDNF is critical for regulating synaptic transmission and plasticity during recovery from these diseases [95]. Depletion of microglia leads to a decrease in synapse-related proteins and glutamatergic signals, which seriously affects synaptic experience-dependent plasticity [92]. These findings provide evidence that propofol-induced prevention of neurological damage activates the BDNF/TrkB pathway to promote anti-inflammatory M2 phenotype microglial polarization and exerts anti-inflammatory effects through its downstream PI3K/Akt signaling pathway [96].

### 4.2 Ketamine

Ketamine is a non-competitive NMDA receptor antagonist often used in pediatric anesthesia. Ketamine was found to reduce LPS-induced calcium levels and CaMKII phosphorylation, thereby inhibiting NMDA receptor activation-mediated inflammation in BV 2 cells [56] (Fig. 3).

Ketamine can effectively inhibit inflammation by reducing the expression of pro-inflammatory factors. Ketamine inhibited the LPS-induced increases in TNF-α, IL-1β, and nitrite in primary cultured microglia in a dose-dependent manner, which may be the result of inhibition of ERK1/2 phosphorylation [97]. In a rat model of post-traumatic stress disorder, esketamine reduced pro-inflammatory cytokines, NF-κB phosphorylation, and microglial activation in the dorsal striatum and periaqueductal gray [98]. In contrast to the above findings, ketamine also has pro-inflammatory effects. In vitro studies have confirmed that ketamine induces polarization of microglia toward a pro-inflammatory M1 phenotype, which enhances the neurotoxicity of ketamine in neurons [36]. It is currently believed that ketamine-induced neuroinflammation is associated with high doses and prolonged exposure and that multiple and prolonged exposures to ketamine will increase hippocampal IL-6 and IL-1β levels in a dose-dependent manner. Mice receiving 60 mg/kg of ketamine administered for 6 months showed spatial memory deficits, whereas there were no significant deficits at lower doses [99]. These findings are supported by another study in which rats exhibited learning and memory deficits when exposed to 80 mg/kg rather than 30 mg/kg of ketamine [100]. In addition, a single clinically relevant dose of ketamine (7 mg/kg) can induce an inflammatory reaction and cognitive impairment in the hippocampus of developing rats, which is related to activation of the NLRP3 inflammatory body/caspase-1 axis [101, 102]. These findings further suggest a susceptibility to the pro-inflammatory effects of ketamine during an age-related window of vulnerability. Therefore, there must be tight quality control of the ketamine dose and timing and the age of subjects if it is to be used clinically to prevent or alleviate PND.

A link exists between the anti-inflammatory mechanisms of ketamine and its rapid antidepressant effects. Ketamine inhibited increases in TNF-α, IL-1β, and IL-6 levels in the brains of stressed mice and reduced depression-like behavior. This result was closely related to the number of reactive microglia and the TLR4/p38 MAPK pathway [103]. Ketamine inhibits microglial activation through the MAPK ERK1/2 pathway [97]. Ketamine-induced phosphorylation of the ERK pathway is essential in the production of BDNF and anti-inflammatory cytokines. The regulation of BDNF gene expression by the ERK-NRBP 1-CREB pathway in microglia provides a foundation for the antidepressant effects of ketamine [104]. Ketamine and esketamine, as NMDA receptor blockers, induce glutamate binding to AMPA receptors, which then triggers BDNF release and inhibits NF-κB translocation via the TrkB pathway, through a downstream intracellular cascade, to reduce postoperative depressive behavior [105, 106]. Regarding neuropathic pain, BDNF secreted by microglia can restore cortical plasticity and reduce hyperalgesia [107]; therefore, the facilitation of BDNF by ketamine in microglia explains the analgesic effect of ketamine to some extent.

Ketamine can regulate the type I interferon pathway through nuclear translocation of STAT-3 in human microglia [108]. Conversely, STAT-3, an important transcription mediator of pro-inflammatory cytokines, may interact with eukaryotic elongation factor 2, leading to an increase in BDNF, thus participating in the antidepressant effect of ketamine. In addition, in the inflammatory state, microglia participate in the tryptophan metabolism pathway, which leads to quinolinic acid production. Quinolinic acid has an inflammatory effect as an NMDA receptor agonist [109]. Ketamine can play an antidepressant role by inhibiting quinolinic acid and increasing kynurenic acid production [110]. These findings show that NMDA receptors in microglia play an important role in anti-depressive effects.

RAGE is an important PRR for inflammation [111]. As a DAMP molecule, high mobility group box 1 can recognize TLRs and RAGE in microglia and mediate the expression of pro-inflammatory mediators through the canonical NF-κB pathway [112]. Studies have shown that ketamine administration in mice can decrease the polarization of pro-inflammatory microglia by inhibiting the high-mobility group box 2/RAGE/NF-κB axis induced by LPS (Fig. 3). This not only relieves inflammation but also has an antidepressant effect [113]. These findings indicate that ketamine can prevent or treat depression by reducing the accumulation of high mobility group box-1 and RAGE, thereby providing new therapeutic targets.

### 4.3 Sevoflurane

Sevoflurane is the most commonly used volatile anesthetic agent in clinical practice. It exhibits both pro-inflammatory and anti-inflammatory effects (Fig. 3). GABA-receptive microglia selectively modify inhibitory synapses during neurodevelopment, and excessive activation or disruption of GABAB receptors in microglia can lead to behavioral abnormalities [114]. Sevoflurane, a GABA receptor agonist, is involved in GABAB receptor-mediated inhibition and may promote behavioral abnormalities (Fig. 3). GABA induces an inflammatory response in microglia in a concentration- and time-dependent manner consistent with the dual anti-inflammatory and pro-inflammatory effects produced by GABAergic anesthetics [115]. The onset of PND in aging mice is related to BDNF/TrkB pathway signaling abnormalities and is mediated by the NMDA receptor/calcium/calpain pathway [116]. Because sevoflurane targets NMDA receptors and increases the intracellular calcium concentration, it is reasonable to suggest that sevoflurane induces PND in older individuals via this crosstalk pathway.

Many in vivo and in vitro studies have shown that inhalational anesthetics can activate the NF-κB signaling pathway, resulting in pro-inflammatory effects [37]. Sevoflurane can exacerbate nerve damage by promoting activation of the pro-inflammatory M1 phenotype (2%, 5 h) and inhibiting the anti-inflammatory M2 phenotype in microglia (2% or 4%, 12 h) [38, 117, 118]. Exposure of rats to sevoflurane (3%, 2 h) induced hyperactivation of NF-κB signaling and led to cognitive dysfunction [119, 120]. In addition, sevoflurane exposure in neonatal (3%) and geriatric stages (3.6%), but not in adulthood, caused neuroinflammation and cognitive impairment [121, 122] (Table 2). Thus, cognitive deficits induced by sevoflurane exposure may be dependent on the developmental stage, anesthetic dosage, duration of exposure, and number of exposures. Although its neurotoxicity has been widely discussed, sevoflurane still has some neuroprotective effects. PI3K is an intracellular lipid kinase that can be activated by binding of its regulatory subunits to adapter proteins. Adapter proteins in turn bind to receptors located on the surface of microglia including TLRs and TrkB [123]. In a PND model of surgical anesthesia for repair of tibial fractures, activation of TLRs triggered GSK-3β, which is phosphorylated via the PI3K/AKT signaling pathway (Fig. 3). This action then inhibited activation of the NF-κB signaling pathway in microglia, decreasing the M1 phenotype and increasing the M2 phenotype [124]. The currently known protective effects of sevoflurane are mainly based on ischemic models. Subanesthetic doses of sevoflurane after pretreatment (2.5%, 1 h, 5 days) promoted activation of M2-phenotype anti-inflammatory microglia through Nrf2 nuclear translocation mediated by GSK-3β phosphorylation, which participates in the neuroprotective effect against cerebral ischemia [40]. Similarly, in an I/R model, sevoflurane reduced the inflammatory response in the infarct zone by inhibiting mRNA and protein expression of TLR4 and NF-κB p65 in the TLR4/NF-κB pathway [125]. In inflammatory situations, the use of a low dose of sevoflurane (2%) attenuated LPS-mediated neuroinflammation as well as neuronal loss, thereby improving spatial recognition deficits [126]. These studies suggest that low-dose exposure to sevoflurane is a key factor in its neuroprotective effects. Further exploration of safe thresholds for sevoflurane use would be beneficial to improve the quality of anesthesia.

Tau is a microtubule-associated protein, and its phosphorylation, aggregation, and diffusion are closely linked to neurodegeneration mechanisms in the aging brain [127]. Microglial NF-κB has an irreplaceable position in driving the diffusion and toxicity of tau in mouse models of tauopathy [128]. A recent study found that sevoflurane induces tau or p-tau migration into microglia via EVs, activating the NF-κB signaling pathway [129]. In turn, nuclear factor kappa-light-chain-enhancer of NF-κB signaling drives microglia-induced proliferation and toxicity of tau, leading to the production of inflammatory factors that further exacerbate neuroinflammation and cognitive impairment [127]. Studies have confirmed that microglia can phagocytose tau-containing neurons or synapses with tau pathology via the classical complement pathway, phagocytose secreted tau oligomers, and transfer them to neurons via EV secretion [130]. However, neurons activate microglia via tau, producing neuroinflammatory molecules and cytokines, thus creating a vicious cycle. Sevoflurane, therefore, seems to exacerbate tauopathy by promoting this vicious cycle.

Activation of the NLRP3 inflammasome is followed by assembly in microglia. Its age-dependent activation may lead to age-related cognitive dysfunction [131]. Sevoflurane appears to increase the assembly of NLRP3 inflammatory vesicles, resulting in an aberrant activation process, which induces hippocampal inflammatory responses that mediate cell death by upregulating the NLRP3 vesicle pathway, causing caspase-1 cleavage and downstream IL-1β release (Fig. 3). Activation of NLRP3 is an upstream signal for tau hyperphosphorylation and aggregation that induces tau pathological processes in a dependent manner [132]. Studies have shown that sevoflurane promotes tau pathological processes and induces cognitive impairment, which may be related to the assembly or migration of EVs [133]. Interestingly, temperature control in aged mice eliminated anesthesia-induced hyperphosphorylation of tau and partially reversed the resulting cognitive impairment [134]. These results may provide ideas for the prevention of clinically relevant cognitive impairment.

### 4.4 Isoflurane

Similar to sevoflurane, isoflurane also has a significant pro-inflammatory effect. Microglial physiological function is closely related to calcium signals. Harmful stimuli, such as LPS and hypoxia, can trigger an increase in calcium, which is related to the synthesis and secretion of cytokines and effectors such as reactive oxygen species and nitric oxide in microglia [135]. Treatment of glioma cells (H4 cells) with 2% isoflurane significantly increased the cytoplasmic calcium level and induced inflammation [37]. Furthermore, inhalational anesthetics can induce abnormal calcium release in the endoplasmic reticulum by overactivating IP3 receptors, thus aggravating cell damage [136]. Long-term exposure to isoflurane (0.75%, 6 h) promoted the activation of pro-inflammatory M1-phenotype microglia, which was mediated by the TLR4/NF-κB pathway [137]. Isoflurane exposure (1.5%, 4 h) also induced excessive activation of NF-κB signaling, which led to cognitive dysfunction in rats (Fig. 3) [119, 120]. Similar to sevoflurane, the toxic effect of isoflurane is not only dose-and time-dependent, but is also related to the number of exposures. During brain development, repeated isoflurane exposure will cause greater long-term cognitive impairment than that resulting from a single exposure [138].

Recent studies have shown that TREM2 on microglia may also act as an upstream target of PI3K/Akt signaling, exhibiting anti-neuroinflammatory, anti-oxidative stress, and anti-apoptotic properties in neurons [139]. Isoflurane exposure (3%, 24 h) significantly reduced TREM2 expression on BV2 microglia and promoted microglial activation and production of inflammatory factors [140]. Therefore, targeting this pathway may improve neurological dysfunction as well as treatment of PND and neurodegenerative diseases. In addition, the NLRP3 pathway is important in isoflurane-induced neuroinflammation. Isoflurane induces hippocampal inflammatory responses and cognitive deficits through activation of the NLRP3/caspase-1 axis [101, 102].

Isoflurane is also neuroprotective and can alleviate neuroinflammatory responses by inhibiting microglial activation. Isoflurane (1.1%/2.2%, 30 min) improved neurologic function and reduced infarct size in a brain I/R model [141]. Activation of pro-inflammatory microglia and the inflammatory response were important mechanisms leading to neurological injury in an electromagnetic pulse model. Exposure to Isoflurane (2%, 30 min) promoted the conversion of microglial cells to an anti-inflammatory phenotype by upregulating the levels of SOCS-1 and the NF-κB inhibitor IκB-α, resulting in amelioration of an electromagnetic pulse-induced injury [142]. Thus, low-dose and short-duration pretreatment with isoflurane has a protective effect, but the current study focused on the brain I/R model. Further validation in other disease models is needed.

## 5. Potential Neuroprotective Strategies

The neuroprotective effects of GAAs depend on age and brain pathology. Animals in juvenile and geriatric stages, but not adult stages, are sensitive to GAA exposure, which can inhibit brain neurodevelopment and promote development of PND. Therefore, additional care should be taken in clinical practice to enhance anesthesia management in the young and elderly. Neuroinflammation can be potentially mitigated in patients with neuroinflammatory disorders through the increased use of anti-inflammatory anesthetics, such as moderate doses of ketamine and propofol. Currently, the main focus on the protective effects of inhaled anesthetics has involved models of cerebral ischemia, where pretreatment with sevoflurane and isoflurane inhibit microglial activation via the TLR4/NF-κB pathway and induce polarization of microglia to the M2 phenotype via activation of Nrf2 and inhibition of NF-κB (Table 2). This may be achieved by inhibiting pro-inflammatory microglia infiltration and migration of damaged neurons as well as promoting phagocytosis of cellular debris and secretion of trophic factors by anti-inflammatory microglia [143]. However, the neuroprotective effects exerted by GAAs are closely related to the dose and duration of exposure. Both intravenous and inhalational anesthetics contribute to the reduction of neuroinflammation in single, low-dose, short-term exposures. In addition, certain GAAs have been shown to have neuroprotective properties. For example, the MAPK pathway plays an important role in pro-inflammatory cytokine expression by inducing activation of the transcription factor AP-1, which in turn regulates the pro-inflammatory genes COX2 and NOS2 [144]. However, dexmedetomidine inhibits the MAPK signaling pathway to ameliorate delirium by decreasing IL-6 and IL-8, which are associated with delirium. It also decreases production of the proinflammatory factor COX2, which is associated with impaired memory. Thus, selection and modulation of clinically useful GAAs may benefit patient recovery and prognosis, mitigate adverse postoperative outcomes, and decrease healthcare costs and mortality [145].

Targeting multiple inflammatory pathways activated by microglia is expected to be a useful method to attenuate the neurotoxic effects induced by GAAs. Because of their role in modulating the neuroinflammatory environment and regulating neuronal networks, microglia are emerging as a promising pharmacological target for the treatment of many CNS disorders, including mood disorders, schizophrenia, and neurodegenerative diseases [146]. Numerous approaches to target microglia function exist and may provide translatable therapies for brain-related diseases. These approaches include classical pharmacological agents (non-steroidal anti-inflammatory drugs, melatonin), targeting of microglia receptors (histamine, acetylcholine receptors) or neuron-microglia crosstalk pathways, and ablation of gene-induced microglia depletion. In addition, Kv1.3 channels have been widely discussed as a novel therapeutic target for the prevention of PND [147].

## 6. Challenges and Future Directions

Despite advances in understanding the interactions of GAAs with microglia, several limitations and shortcomings remain. Targeting microglial activation pathways induced by GAAs is expected to be important for preventing and treating PND. However, the specific state of microglia is complex because of their substantial heterogeneity. This leads to differences in various types of microglia in regard to their response to injury, cell morphology, gene expression, and release of soluble factors [148]. In addition, microglial functional alterations are closely related to their location in the brain and the timeline of injury [149]; therefore, relevant experiments require long-term follow-up. These issues pose challenges for the exploration of potential therapeutic strategies.

In addition, current studies confirm differences in microglia responses between rodents and humans. Animal models have demonstrated that GAAs interfere with microglial activation pathways, attenuating PND. However, human positron emission tomography imaging evidence suggests that microglia exhibit an acutely inhibitory phenotype after anesthesia and surgery, rather than the reactive phenotype seen in animal models, but that GAAs still contribute to this process [150]. To better understand the pharmacological mechanisms and microglia functional status induced by GAAs, long-term follow-up after surgery is needed, and the search for more specific microglia-activating ligands in humans would greatly facilitate the practice of anesthesiology. Additionally, single-cell RNA sequencing of animal models before and after anesthesia will facilitate the characterization of microglial activation and the associated signaling pathways, which may contribute to the discovery of therapeutic strategies. The increasing use of human induced pluripotent stem cell experiments will help translate basic experimental results into the clinical setting and provide a preventive mechanism for anesthesia-induced adverse effects.

Indeed, microglia do not exist in isolation and are closely associated with astrocytes. Evidence suggests that astrocytes, as the most abundant type of microglia in the brain, play an important role in PND to attenuate GAA-induced behavioral deficits by releasing gliotransmitters and neurotrophic factors. The cytokines IL-1α, TNF, and C1q, which are released after microglial activation, can induce the activation of A1-specific astrocytes [151]. A1 astrocytes lose their normal functions in synaptic plasticity and promoting neuronal growth and development and participate in neuron and oligodendrocyte death and neurodegenerative diseases. In the early pathological stage of PND, microglial activation plays an irreplaceable role in long-term synaptic inhibition induced by etomidate. This microglial inhibition is associated with cognitive improvement [152]. In contrast, in late PND stages, microglia-activated A1 astrocytes have been shown to induce long-term synaptic inhibition and cognitive deficits. These results suggest that microglia-astrocyte crosstalk plays a key role in the mechanisms of PND. Microglial activation exacerbates neuronal impairment and PND by directly or indirectly modulating astrocyte function. Exploration of networks related to these specific roles will facilitate progress in neurological studies.

## 7. Conclusion

GABA and NMDA receptors expressed in microglia are targets of GAAs. Binding to these receptors induces altered calcium signaling and phosphorylation of calmodulin in microglia, mediating downstream inflammatory signaling. TLRs, TREM2, and RAGE expressed on the surface of microglia are important targets of neuroinflammation, and GAAs affect inflammation by acting on these receptors. The NF-κB signaling pathway is the key pathway involved in neuroinflammation. Both propofol and ketamine demonstrate a neuroprotective effect by inhibiting this pathway. In vivo and in vitro studies have shown that inhalational anesthetics can activate the NF-κB signaling pathway, resulting in a pro-inflammatory effect. Interestingly, in a brain I/R injury model, inhalational anesthetics could inhibit activation of pro-inflammatory phenotype microglia, which may be attributed to the low dose and short time of exposure. The pro-inflammatory effect of repeated and long-term propofol exposure is closely related to downregulation of the BDNF/TrkB/PI3K/Akt pathway and upregulation of NF-κB and the NLRP3 inflammatory corpuscle pathway. The anti-inflammatory effects possessed by ketamine and its derivatives are closely related to their antidepressant properties, which are mediated through the BDNF/TrkB pathway. As a key signal of inflammation, the NLRP3 inflammasome seems to be the common pathway through which GAAs induce a pro-inflammatory effect on microglia. GAAs can release intercellular communication substances and inflammatory mediators by affecting EVs of microglia as well as HCs and GJs, both alleviating and aggravating neurotoxicity. GAAs also interfere with microglial activation by directly affecting microRNA expression.

In recent years, the effects of GAAs on microglia have attracted the attention of researchers. In this review, we have focused on the potential mechanisms of neurotoxicity related to GAA interactions with neurons via microglia. We have analyzed clinical and animal studies that correlate microglia with general anesthesia, hoping to provide guidance for future laboratory and clinical studies. It is our goal that someday clinical anesthesiologists can select appropriate GAAs on the basis of the properties, expected dose, exposure time, and patient conditions to minimize some of the adverse effects related to the use of GAAs.
